# Body connection mediates the relationship between traumatic childhood experiences and impaired emotion regulation in borderline personality disorder

**DOI:** 10.1186/s40479-021-00157-7

**Published:** 2021-05-17

**Authors:** Marius Schmitz, Katja Bertsch, Annette Löffler, Sylvia Steinmann, Sabine C. Herpertz, Robin Bekrater-Bodmann

**Affiliations:** 1 Department of General Psychiatry, Center for Psychosocial Medicine, University of Heidelberg, Heidelberg, Germany; 2 Department of Psychology, LMU Munich, Munich, Germany; 3 Department of Cognitive and Clinical Neuroscience, Central Institute of Mental Health, Medical Faculty Mannheim, Heidelberg University, Mannheim, Germany; 4 Department of Psychosomatic Medicine and Psychotherapy, Central Institute of Mental Health, Medical Faculty Mannheim, Heidelberg University, Mannheim, Germany

**Keywords:** Adverse childhood experiences, Borderline personality disorder, Emotional dysregulation, Interoception, Dissociation, Invalidation, Mind-body connection

## Abstract

**Background:**

Previous studies revealed an association between traumatic childhood experiences and emotional dysregulation in patients with borderline personality disorder (BPD). However, possible mediating pathways are still not fully understood. The aim of the present study was to investigate the potential mediating role of body connection, describing the awareness of the relationship of bodily and mental states, for the association between a history of traumatic childhood experiences and BPD core symptomatology.

**Methods:**

One-hundred-twelve adult female individuals with BPD and 96 healthy female controls (HC) were included. Impaired emotion regulation, traumatic childhood experiences, and BPD symptomatology were assessed with self-report questionnaires. The Scale of Body Connection was used to assess two dimensions of body connection, that is *body awareness*, describing attendance to bodily information in daily life and noticing bodily responses to emotions and/or environment and *body dissociation*, describing a sense of separation from one’s own body, due to avoidance or emotional disconnection. Mann-Whitney U tests were employed to test for group differences (BPD vs. HC) on the two SBC subscales and associations with clinical symptoms were analyzed with Spearman correlations. We performed mediation analyses in the BPD group to test the assumption that body connection could act as a mediator between a history of traumatic childhood experiences and emotion dysregulation.

**Results:**

Individuals with BPD reported significantly lower levels of body awareness and significantly higher levels of body dissociation compared to HC. Body dissociation, traumatic childhood experiences, and emotion dysregulation were significantly positively associated. Further analyses revealed that body dissociation, but not body awareness, significantly and fully mediated the positive relationship between traumatic childhood experiences and impaired emotion regulation in the BPD sample. This mediation survived when trait dissociation, i.e., general dissociative experiences not necessarily related to the body, was statistically controlled for.

**Conclusion:**

Certain dimensions of body connection seem to be disturbed in BPD patients, with body dissociation being an important feature linking a history of traumatic childhood experiences to current deficits in emotion regulation.

**Supplementary Information:**

The online version contains supplementary material available at 10.1186/s40479-021-00157-7.

## Background

Emotion dysregulation represents one of the core features of borderline personality disorder (BPD), which includes deficits in the recognition and control of own emotions [1] and which has been empirically and etiologically associated with traumatic childhood experiences [2–6]. Traumatic childhood experiences before the age of 18 comprise different categories such as emotional and physical abuse and neglect, sexual abuse, and a dysfunctional parental home, such as living with family members displaying substance abuse [7, 8]. According to etiological models of BPD [9, 10], consistent invalidation by primary caregivers plays an important role in the development of borderline behavior patterns in emotionally vulnerable individuals. Growing up in an invalidating environment involves the perpetual feedback that emotional experiences and expressions are not deemed to be appropriate responses [9]. Consequences as stated by common etiological models [11] include emotional dysregulation as neurobiological disposition which manifests due to invalidation [9], failed mentalization as the inability to identify mental states in oneself and others and their interactions due to inadequate mirroring by primary caregivers [10], self- and other-directed aggression either due to genetically determination or excessive frustrations during childhood [12], and interpersonal hypersensitivity, which, according to Gunderson’s gene-environment-developmental model [13], might reflect a genetic disposition to react to perceived failures of social support with maladaptive behaviors such as dissociation or impulsivity. Despite some important differences, the overlap of these models is the assumed role of early experiences on emotion regulation. Therefore, traumatic childhood experiences, such as emotional neglect and abuse, might impact learning of the regulation of one’s own emotions in affected children: *emotional neglect* has been defined as a failure to meet children’s basic emotional and psychological needs, while *emotional abuse* consists of verbal assaults or any humiliating or demeaning behavior by an adult or older person [7]. Studies suggest that emotional abuse and emotional neglect, compared to other forms of traumatic childhood experiences, seem to be particularly associated with BPD [14].

There is growing evidence that awareness of and a sense of connectedness to one’s own body might be an important mediator for the observed link between traumatic childhood experiences and emotion dysregulation in BPD [15, 16]. Patients with BPD show perceptual and verbal deficits regarding their own emotions [17], difficulties in using appropriate emotion regulation strategies [18], and first evidence suggests a reduced cortical representation of physiological processes from the inner body in BPD [19, 20]. The finding that patients with BPD rely more on external emotional cues for emotion recognition [21, 22] suggests that patients with BPD might put less trust in their own body responses as cues for their own emotional experiences. However, prominent theories such as the “somatic marker hypothesis” [23] suggest that the acquisition of adequate emotional responses requires ongoing perception and interpretation of physiological processes [24], which underlines the importance of an intact body connection, that is, a state of observational *body awareness* and acceptance of body experiences opposed to *body dissociation* [25]. Body awareness and body dissociation have been empirically identified as independent dimensions of body connection, representing different aspects of being aware of the relationship between bodily and mental states [25]. While *body awareness* subsumes the perception of inner physiological processes entering one’s consciousness and the willingness to attend to those inner signals for self-care [26], *body dissociation* describes a non-pathological detachment from one’s body in an attempt to avoid adverse body experiences [25]. Body dissociation ranges from distraction from bodily experiences to feelings of detachment from one’s own body and emotional disconnection, which overlaps with, but is not identical to, other dissociative experiences [27]. For instance, psychoform dissociation ranges from mild forms, such as daydreaming and absorption, to severe forms that are frequently reported by individuals with BPD [28–30], and somatoform dissociation refers to physically manifested dissociative symptoms, including hyposensitivity for pain, a highly prevalent feature in individuals in BPD [31]. Besides altered physical domains such as pain perception, altered body perception has been related to psychoform dissociation in BPD, including enhanced body plasticity in terms of a disposition to accept a non-body object as part of the own body and reduced body ownership experiences in terms of a perceived foreignness of the own body [32, 33]. Therefore, both psychoform and somatoform dissociation represent disruptions of body connection which have been shown to covary and to occur in individuals with a history of traumatic childhood experiences [34, 35] and under stress in BPD [36–38]. In addition to psychoform and somatoform dissociation, body dissociation in its current definition can be seen as a coping style and inner attitude toward one’s own body, which might be altered due to traumatic childhood experiences and be further enhanced by current dissociative states.

Although current models and first empirical findings suggest that deficient body connection could be an important mediator for the relationship between traumatic childhood experiences (particularly emotional neglect and abuse) and emotion dysregulation, this assumption has not yet been specifically adressed. In the present study, we thus used the Scale of Body Connection (SBC) [25, 26] to assess body connection in a large sample of female patients with BPD in comparison to age- and sex-matched healthy controls (HC). We expected reduced body connection, i.e., lower body awareness and higher body dissociation, in BPD patients compared to HC. Participants were further asked to fill the *Childhood Trauma Questionnaire* (CTQ) and the *Deficits in Emotion Regulation questionnaire* (DERS), for which we performed mediation analyses, adding body connection measures as mediators. Due to the crucial role of emotional abuse and neglect for the etiology of BPD, we particularly focused on these subscales of the CTQ. Furthermore, we included trait dissociation measured with the German version of the *Dissociative Experience Scale* as a control variable due to its overlap with body dissociation as a component of body connection.

## Methods

### Design

This research was part of a larger study cohort recruited by the central office of the KFO 256, a Clinical Research Unit funded by the German Research Foundation (DFG) for investigating the mechanism of disturbed emotion processing in BPD [39]. All participants gave written informed consent before study participation and provided demographical data and clinical self-reports. A two-group cross-sectional design was employed. The study was approved by the ethics review board of the Medical Faculty Mannheim, Heidelberg University, and adhered to the Declaration of Helsinki in its current form.

### Recruitment and enrollment

Participants with BPD were recruited from online announcements, flyers, and the pool of in- and out-patients of the Department of Psychosomatic Medicine and Psychotherapy at the Central Institute of Mental Health and of the Department of General Psychiatry at the University of Heidelberg. HC were recruited through the local resident’s registration office. Recruitment of all participants in our study was undertaken by the central office of the KFO 256. Hence, all projects linked to the KFO 256 included participants from a joint database. Trained psychologists with at least a master’s degree conducted the assessments of both patients and HC. The diagnosis of BPD according to DSM-IV [36] was assessed with the International Personality Disorder Examination interview (IPDE) [40]. Other psychiatric diagnoses were assessed with the SCID-I for Axis I disorders [41]. All participants were fluent in the German language.

Inclusion criteria for the BPD group were five or more IPDE criteria a) at least over a period of the last 5 years including the last 12 months (current BPD) or b) once during their life (remitted BPD). Inclusion criteria for the HC group were a) no current or lifetime psychiatric diagnosis and b) no current or lifetime psychological/psychiatric treatment. General exclusion criteria for all participants within the KFO 256 consisted of a) neurological disorders, b) severe illness, c) pregnancy, d) current alcohol or drug abuse or e) substance dependence in the last 2 months, f) lifetime diagnosis of schizophrenia, schizoaffective or bipolar-I disorder, and g) medication, except for selective serotonin reuptake inhibitors (SSRIs), as SSRIs are often used to treat anxiety disorders and depression commonly co-occurring with BPD [42, 43].

Although not in the disorder’s current state, remitted BPD patients have been proved to show persistent emotion regulation deficits [44, 45]. The patient sample therefore included individuals with a current diagnosis of BPD (*n* = 94) as well as those with BPD in remission (*n* = 18).

### Assessments

The *Scale of Body Connection* (SBC) [25] assesses the two independent dimensions *body awareness* and *body dissociation* during the last 2 months. *Body awareness* (12 items; overall internal consistency in the present study of (Cronbach’s alpha) *α* = .77 for the patient group (BPD) and *α* = .79 for the healthy control group (HC)) measures attention to bodily signals in everyday situations and the perception of bodily responses to emotions (e.g. “I notice that my breathing becomes shallow when I am nervous”). *Body dissociation* (8 items; overall internal consistency in the present study *α* = .79 (BPD) and *α* = .63 (HC)) refers to the avoidance or disregard of internal bodily experiences and the feeling of seperatedness from one’s own body (e.g. “I distract myself from feelings of physical discomfort”). Each item is scored on a 5-point scale, ranging from 0 ‘not at all’ to 4 ‘all of the time’. Scale values depict mean scores across the 12 and 8 items, respectively. Each scale includes a question about body connection during sexual activity which can be left blank if the participant has not been sexually active in the last 2 months, including self-stimulation (which was the case in 4,8% of participants; missing values were omitted for calculating the mean scores). Mean scores ranged from 0 to 4 with higher values indicating higher body awareness and body dissociation, respectively. A German translation of the SBC, based on its original English version [22], was used in the present study (unpublished).

Traumatic childhood experiences were assessed with the *Childhood Trauma Questionnaire* (CTQ) [7], which has been shown to be reliable and valid. Participants were asked to rate the frequency of traumatic experiences on a 5-point scale (ranging from ‘never true’ to ‘very often true’) for the five scales *physical* (e.g. ‘People in my family hit me so hard that it left me with bruises or marks’), *sexual* (e.g. ‘Someone molested me’), and *emotional abuse* (e.g. ‘I thought that my parents wished I had never been born’), and *physical* (e.g. ‘I had to wear dirty clothes’) and *emotional neglect* (e.g. ‘I felt loved’ [reverse coded]) with five items each (resulting in corresponding scores from 5 to 25). A total sum score was calculated from the scales [7], ranging from 25 to 125 (overall internal consistency in the present study *α* = .88 (BPD) and .69 (HC)), with higher values indicating a higher frequency of traumatic experiences.

Deficits in emotion regulation were assessed with the *Difficulties in Emotion Regulation Scale* (DERS) [46]. The DERS comprises six subscales: *nonacceptance of negative emotions* (6 items; e.g. ‘When I’m upset, I feel like I am weak’), *difficulties engaging in goal-directed behaviors when distressed* (5 items; e.g. ‘When I’m upset, I have difficulty getting work done’), *difficulties controlling impulsive behaviors when distressed* (6 items; e.g. ‘I experience my emotions as overwhelming and out of control’), *limited access to effective emotion regulation strategies* (8 items; e.g. ‘When I’m upset, I believe that I’ll end up feeling very depressed’), *lack of emotional awareness* (6 items; e.g. ‘When I’m upset, I believe that my feelings are valid and important’ [reverse coding]), and *lack of emotional clarity* (5 items; e.g. ‘I have no idea how I am feeling’) [3]. Participants rated each item on a 5-point scale ranging from ‘almost never’ to ‘almost always’. A total sum score (internal consistency in the present study *α* = .94 (BPD) and .88 (HC)) can be calculated from the scales [46], ranging from 36 to 180, with higher values indicating more severe deficits in emotion regulation.

Trait dissociation was assessed with the German adaptation of the *Dissociative Experience Scale*, that is, the *Fragebogen zur Erfassung Dissoziativer Symptome* (FDS) [47, 48]. The FDS consists of 44 items measuring the frequency of dissociative experiences (in 10% increments, ranging from 0 to 100) on the dimensions *amnesia* (e.g. ‘Some people find evidence that they have done things that they do not remember doing’), *absorption/imaginative involvement* (e.g. ‘Some people have the experience of not being sure whether things that they remember happening really did happen or whether they just dreamed them’), *derealisation/depersonalization* (e.g. ‘Some people sometimes have the experience of feeling that other people, objects, and the world around them are not real’), and *conversion* (e.g. ‘Some people sometimes have difficulties with their eyes (e.g. double or blurred vision, blind in one or both eyes, without a doctor being able to find a physical cause’). The FDS proved to be a reliable and valid screening tool for major dissociative disorders and BPD [32, 49]. In the present study, the FDS mean score was used as a measure of overall trait dissociation (internal consistency in the present study *α* = .94 (BPD) and .90 (HC), ranging from 0 to 100, with higher values indicating higher overall trait dissociation.

In addition, borderline symptom severity was assessed with the short version of the *Borderline Symptom List* (BSL-23; internal consistency in the present study *α* = .94 (BPD) and .86 (HC); mean scores ranged from 0 to 4) [50], depressiveness with the *Beck-Depression-Scale* (BDI-II; internal consistency in the present study *α* = .89 (BPD) and .77 (HC); sum scores ranged from 0 to 63) [51], and trait anxiety with the State-Trait-Anxiety Inventory (STAI) [52] internal consistency in the present study *α* = .93 (BPD) and .89 (HC); sum scores ranged from 20 to 80) [52]. These additional self-reports were administered in order to assess the symptom severity of BPD and further psychopathological features.

### Data analyses

Variables were checked for normal distribution using the Shapiro-Wilk test. Since the assumption of normality was violated in most variables for at least one of the two groups (corresponding values: W ≤ .960, *p* ≤ .002) and transformation of the variables was only insufficiently successful, non-parametric tests models were used for statistical analysis of non-transformed data. Analyses were performed using IBM SPSS v26.0 (descriptives and correlation analyses) and R v3.5.0 via R plug-in for SPSS (mediation analysis).

First, a Mann-Whitney U test was used to test for group differences (BPD vs. HC) in body awareness and body dissociation (*r* as effect size) [53]. Furthermore, associations between body awareness, body dissociation, and other dissociative trait experiences (FDS) were analyzed with Spearman correlations. Additional exploratory correlation analyses are reported in the supplement (see Table S1). Significant correlation coefficients were compared using Fisher’s z-transformation.

Second, the proposed mediating role of body connection was tested in mediation models. The HC group was excluded from the mediation and correlational analyses due to a lack of variance in clinical self-reports and variables of interest: Almost one fifth of HC (19.8%) reported no history of traumatic childhood experiences (indicated by a CTQ score of 25), and 40.6 and 42.7% of the HC reported no experiences of emotional neglect or emotional abuse, respectively, which were entered as predictors in the mediation models. Therefore, only the patient sample was included in the mediation and correlational analyses (however, explorative mediation models for the combined BPD-HC sample can be found in the supplement (see Fig. S3). A mediation model including body awareness and body dissociation as parallel mediators (Model 1; analyses including body awareness and body dissociation as separate mediators are reported in the supplement, see Figs. S4 and S5) using the ROBMED macro with robust bootstrap for SPSS (v0.6.0) [54] (bootstrapping procedure: 10,000 samples, confidence intervals: 95%, unstandardized coefficients, adjusted robust R^2^ as effect size) was computed. Path A in the mediation model represents the basic relationship between the predictor (early traumatization as measured with the CTQ, total score) and each mediator (see Fig. 2A). Path B represents the combined relationship of each mediator with the outcome (deficits in emotion regulation as measured with the DERS total score) with the direct effect representing the effect of the predictor on the outcome after the inclusion of the mediators in the model. The basic relationship between the predictor and the outcome is denoted by the total effect. The indirect effect represents the combined effect of path A and path B and therefore the mediation. Significance inferences at the 0.05 *α* level are based upon the notion whether confidence intervals include zero. In a second step, trait dissociation (as assessed by the FDS total score) was added as covariate (Model 2, see Fig. 2B). In order to give an estimate of somatoform and psychoform dissociation, we additionally conducted separate mediation analyses with the conversion scale of the FDS (which shows high correlation with somatoform dissociation [35]) and the DES as parallel mediators instead of the global FDS score which can be found in the supplement (see Fig. S6).

In a third step, we explored whether the proposed mediation model (Model 2) hold true for subscales of the CTQ, namely *emotional neglect* (see Fig. 3A) and *emotional abuse* (see Fig. 3B). We chose these subscales, as *emotional neglect* and *emotional abuse* involve self-reports of interpersonal emotional disruptive events and have been particularly associated with BPD. For specificity purposes, we further report on the proposed mediation model for the remaining three subscales of the CTQ, namely *physical neglect*, *physical abuse* and *sexual abuse* in the supplement.

## Results

### Sample characteristics

A total of 112 adult female participants with BPD (*M*_age_ = 29.76 ± 7.41 years) and 96 female healthy controls (HC, *M*_age_ = 28.01 ± 7.58 years) were included in the present study (see Table 1 for details). The patient sample included participants with a current diagnosis of BPD (*n* = 94) as well as those with BPD in remission (*n* = 18). The BPD and HC group did not differ in age (*t*_*(207)*_ = 1.68, *p* = .095). Further inferential statistics for clinical and self-reported data are reported in Table 1.

**Table 1:** Clinical and self-reported data of patients and HC

Construct	Mean ± SD (Mdn; IQR)		***U***	***P*** value^**a**^	Cohen’s ***d***
	Patient group (*n* = 112)	HC (*n* = 96)			
**BPD dimensional Score (IPDE)**	12.65 ± 4.22 (14.00; 3.00)	0.09 ± 0.46 (0.00; 0.00)	112.00	<.001	4.034
**Borderline symptoms (BSL-23 Score)**	1.33 ± 0.80 (1.22; 1.33)	0.12 ± 0.19 (0.04; 0.17)	384.00	<.001	2.012
**Depressiveness (BDI)**	18.90 ± 10.25 (18.00; 15.00)	2.10 ± 2.93 (1.00; 3.00)	474.00	<.001	2.159
**Trait-Anxiety (STAI)**	59.18 ± 10.86 (61.00; 13.00)	31.85 ± 7.21 (32.00; 9.00)	358.50	<.001	2.921
**Dissociation (FDS)**	17.89 ± 11.82 (16.59; 14.94)	3.42 ± 3.65 (2.27; 3.64)	978.50	<.001	1.604
**Traumatic childhood experiences (CTQ)**	61.16 ± 18.40 (60.00; 25.25)	30.98 ± 7.65 (28.00; 7.50)	557.00	<.001	2.086
Emotional Abuse	16.96 ± 5.35 (17.00; 8.00)	6.89 ± 2.81 (6.00; 2.75)	527.00	<.001	2.306
Physical Abuse	8.60 ± 4.62 (7.00; 5.75)	5.48 ± 1.89 (5.00; 0.00)	2322.50	<.001	0.860
Sexual Abuse	8.36 ± 5.22 (6.00; 5.00)	5.09 ± 0.54 (5.00; 0.00)	2779.00	<.001	0.852
Emotional Neglect	17.28 ± 5.80 (18.00; 9.00)	7.47 ± 3.16 (7.00; 4.00)	870.50	<.001	2.058
Physical Neglect	9.97 ± 3.80 (9.00; 5.00)	6.05 ± 1.87 (5.00; 1.00)	1751.50	<.001	1.279
**Emotional Dysregulation (DERS Score)**	122.52 ± 25.61 (127.50; 33.50)	65.73 ± 14.80 (63.50; 17.00)	428.00	<.001	2.664

*Abbreviations*: *BDI* Beck Depression Inventory, *BPD* Borderline personality disorder, *BSL-23* Short version of the Borderline Symptom List, *CTQ* Childhood Trauma Questionnaire, *DERS* Difficulties in Emotion Regulation Scale, *FDS* German adaptation of the Dissociative Experience Scale (DES), *HC* Healthy controls, *IPDE* International Personality Disorder Examination, *IQR* Interquartile range, *M* Mean, *Mdn* Median, *P* Value Probability value, *SD* Standard deviation, *STAI* State-Trait-Anxiety Inventory, *U* Test statistic of the Mann-Whitney U test

^a^Uncorrected for multiple testing

The BPD patient sample showed a high number of comorbid disorders including affective disorders (*n* = 27, lifetetime diagnosis: *n* = 90), posttraumatic stress disorder (*n* = 28, lifetetime diagnosis: *n* = 47) and other anxiety disorders (*n* = 47, lifetetime diagnosis: *n* = 62), body dismorphic disorder (*n* = 1, lifetetime diagnosis: *n* = 1) and eating disorders (*n* = 18, lifetetime diagnosis: *n* = 55), as well as antisocial (*n* = 1, lifetetime diagnosis: *n* = 4) and avoidant (*n* = 30, lifetetime diagnosis: *n* = 34) personality disorder. Regarding medication, 13 patients took SSRI. Participants in the HC group neither received any diagnosis of a mental disorder nor took medication.

### SBC group differences

**Fig. 1:**
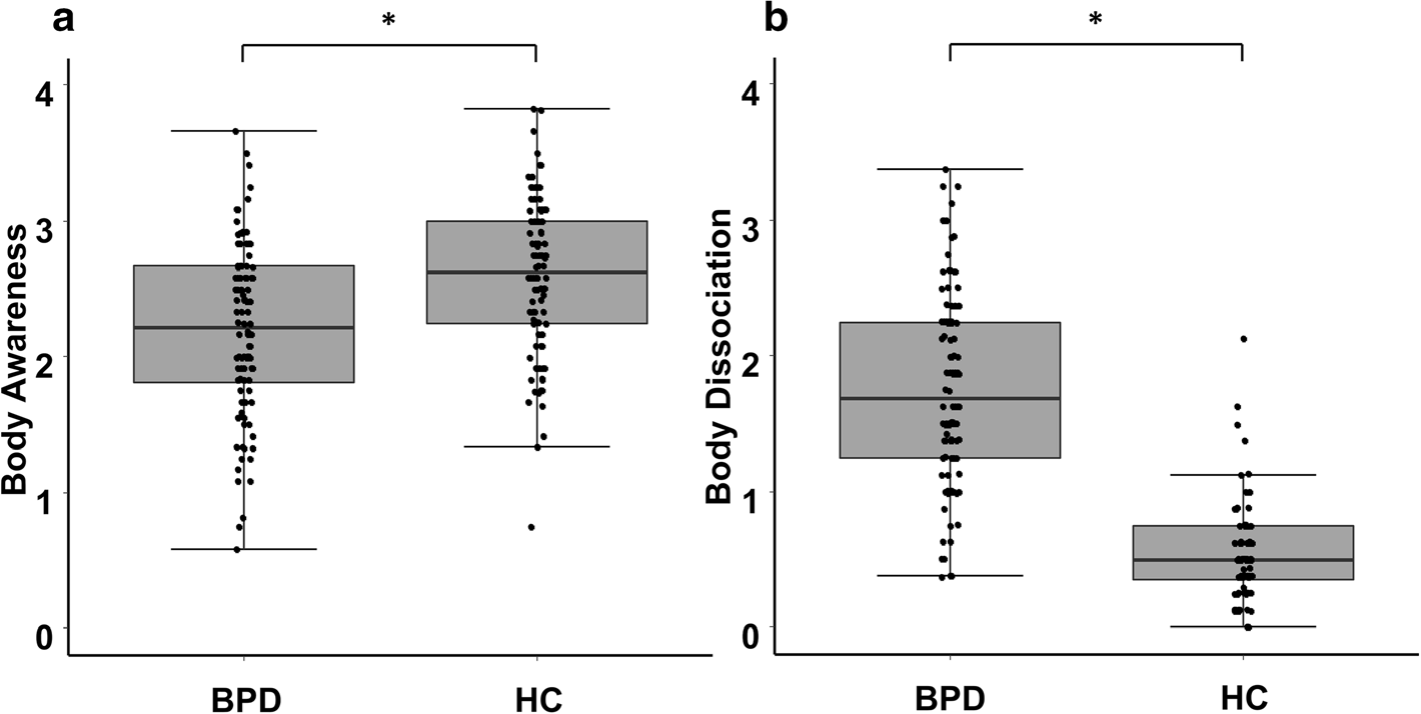
Body connection in the study samples. Given are the boxplots and individual data points for **a**) body awareness and **b**) body dissociation in patients with borderline personality disorder (BPD) and healthy controls (HC). Values above or below than 1.5 * interquartile range are considered to be outliers. * *p* < .001

The groups differed significantly in both *body awareness* and *body dissociation*. As hypothesized, participants with BPD showed lower levels of body awareness (*M* = 2.21; *SD* = 0.61; *Mdn* = 2.22; *IQR* = 0.90) than HC (*M* = 2.59; *SD* = 0.58; *Mdn* = 2.63; *IQR* = 0.75), *U* = 7244.50, *z* = − 4.32, *p* < .001, *r* = .30 (Fig. 1a). Vice versa, participants with BPD showed higher levels of body dissociation (*M* = 1.76; *SD* = 0.70; *Mdn* = 1.69; *IQR =* 1.00) compared to HC (*M* = 0.53; *SD* = 0.61; *Mdn* = 0.50; *IQR* = 0.44), *U* = 622.50, *z* = − 11.00, *p* < .001, *r* = .76 (Fig. 1b). Additional separate results for the subsample of participants with remitted BPD can be found in the Supplemental Material (see Figs. S1 and S2, Tables S2 and S3).

### Correlation analyses

Body awareness was negatively correlated with deficits in emotion regulation (*ρ* = −.231, *p* = .007) and trait dissociation (*ρ* = −.190, *p* = .023) in patients with BPD, whereas body dissociation was positively correlated with deficits in emotion regulation (*ρ* = .392, *p* < .001; comparison of body awareness and body dissociation coefficients: *z* = − 4.30, *p* < .001) and trait dissociation (*ρ* = .551, *p* < .001; comparison of body awareness and body dissociation coefficients: *z* = − 5.35, *p* < .001). However, only body dissociation was significantly correlated with traumatic childhood experiences (*ρ* = .241, *p* = .005), while body awareness was not (*ρ* = −.115, *p* = .114). Since trait dissociation seems to share at least some of the variance with the two dimensions of body connection, we controlled for trait dissociation in the subsequent mediation analyses.

### Mediation analyses

**Fig. 2:**
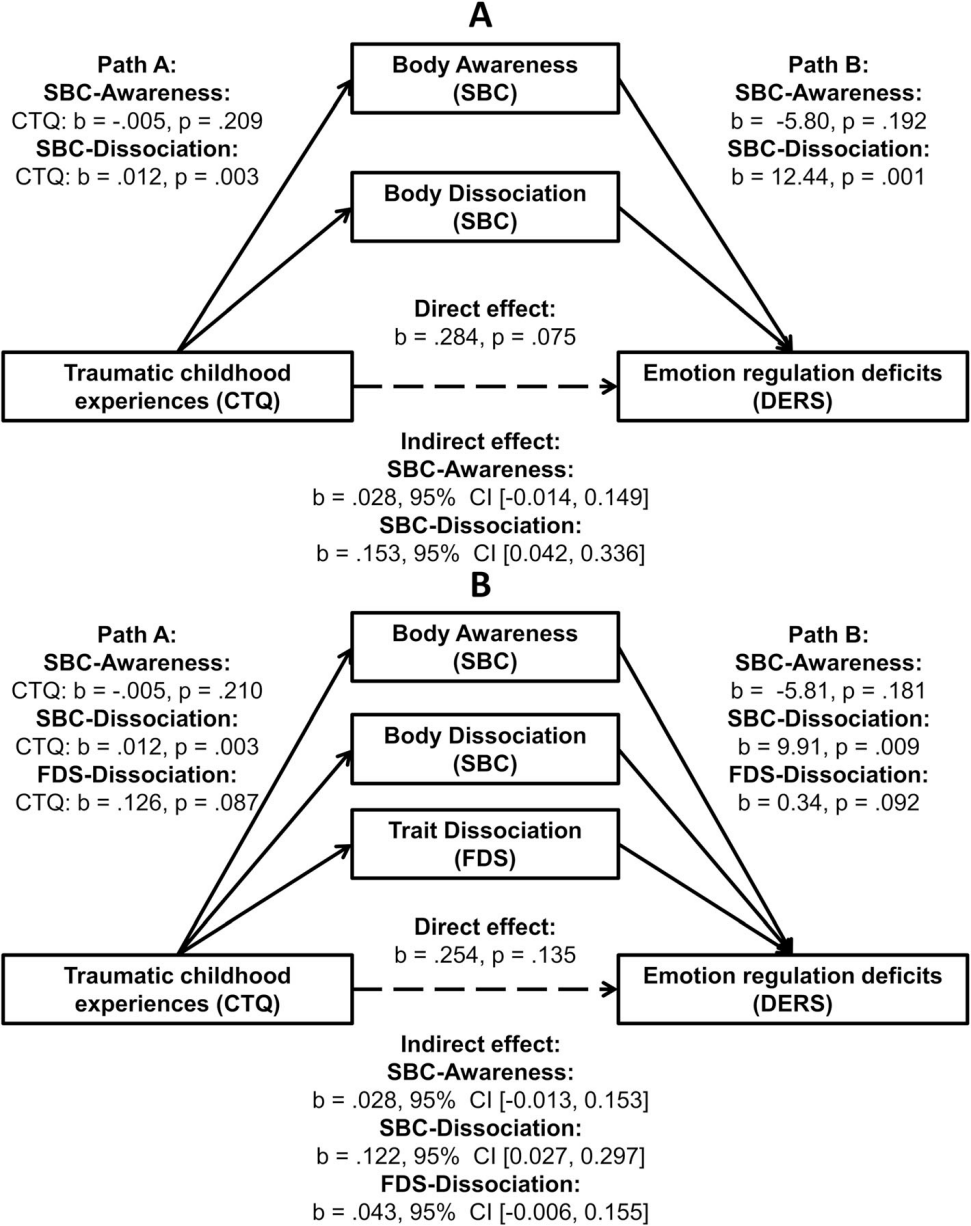
Parallel mediation of early traumatization and emotion regulation deficits by *body awareness* and *body dissociation* in women with BPD. *Path A* represents the effect of early traumatization on each mediator; *Path B* represents the combined effects of each mediator on emotion regulation deficits; the *direct effect* represents the effect of early traumatization on emotion regulation deficits, while keeping levels of the mediators constant; the *indirect effect* represents the combined effect of path A and path B and therefore the mediation. The *total effect* (not shown here) represents the combined indirect and direct effects. Significance inferences at the 0.05 α level for *indirect effects* are based upon the notion whether confidence intervals include zero. Trait dissociation included as parallel mediator in Model B. *Abbreviations:* CTQ, Childhood Trauma Questionnaire; DERS, Difficulties in Emotion Regulation Scale; FDS, German adaptation of the Dissociative Experience Scale; SBC; Scale of Body Connection

There was a significant indirect effect of traumatic childhood experiences (CTQ total score) on emotion dysregulation (DERS total score) through body dissociation (*b* = .153, 95% CI [0.042, 0.336]), but not through body awareness (*b* = .028, 95% CI [− 0.014, 0.149]) in the patients (Model 1; see Fig. 2A). While the total effect of traumatic childhood experiences on emotion dysregulation was significant (*b* = .465, *p* = .003), the direct effect was not statistically significant after including body dissociation and body awareness (*b* = .284, *p* = .075; adjusted robust *R*^2^ = .207). The pattern of indirect, direct, and total effects suggest that body dissociation, but not body awareness, fully mediated the association between traumatic childhood experiences and emotion dysregulation in BPD. Including trait dissociation (FDS total score) as parallel mediator did not change the pattern of results (Model 2; *b* = .122, 95% CI [0.027, 0.297] for body dissociation, *b* = .028, 95% CI [− 0.013, 0.153] for body awareness, and *b* = .043, 95% CI [− 0.006, 0.155] for trait dissociation; see Fig. 2B). Again, the total effect was significant (*b* = .447, *p* = .007), whereas the direct effect was statistically not significant (*b* = .254, *p* = .135; adjusted robust *R*^*2*^ = .216), suggesting that body dissociation fully mediated the association between traumatic childhood experiences and emotion dysregulation in BPD even after controlling for trait dissociation. Additional mediation analyses with body awareness and body dissociation as separate mediators confirmed the above described results and can be found in the Supplemental Material (Figs. S4 and S5).

**Fig. 3:**
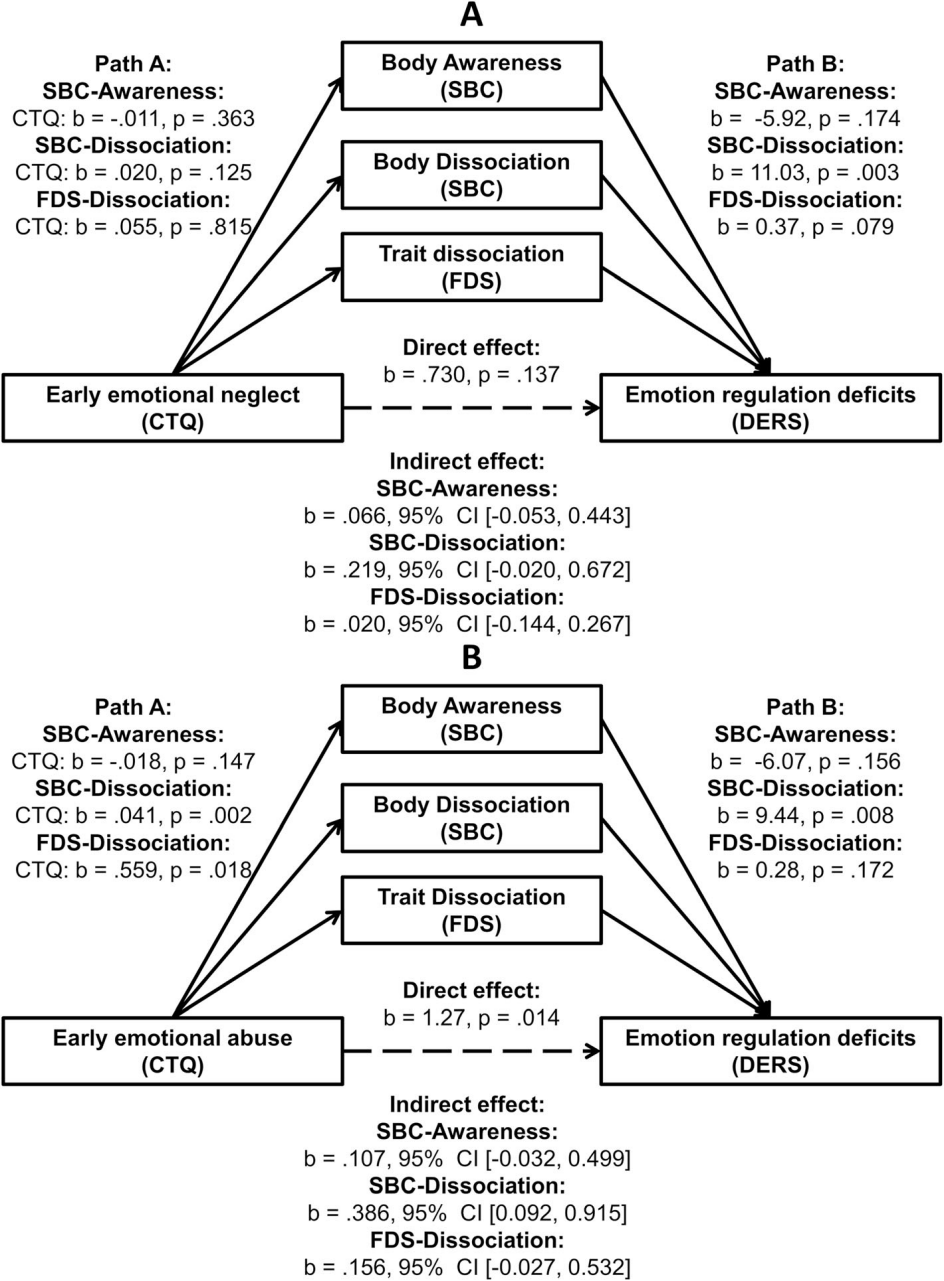
Parallel mediation of early emotional neglect (Model A) and early emotional abuse (Model B) and emotion regulation deficits by *body awareness* and *body dissociation* in women with BPD. *Path A* represents the effect of early traumatization on each mediator; *Path B* represents the combined effects of each mediator on emotion regulation deficits; the *direct effect* represents the effect of early traumatization on emotion regulation deficits, while keeping levels of the mediators constant; the *indirect effect* represents the combined effect of path A and path B and therefore the mediation. The *total effect* (not shown here) represents the combined indirect and direct effects. Significance inferences at the 0.05 α level for *indirect effects* are based upon the notion whether confidence intervals include zero. Trait dissociation included as parallel mediator. *Abbreviations:* CTQ, Childhood Trauma Questionnaire; DERS, Difficulties in Emotion Regulation Scale; FDS, German adaptation of the Dissociative Experience Scale (DES); SBC; Scale of Body Connection

We also explored whether the mediation model held true for the two individual CTQ subscales *emotional neglect* and *emotional abuse*, since these two forms of traumatic childhood experiences are supposed to be most strongly associated with emotion dysregulation in BPD. While the indirect effect of emotional neglect on emotion dysregulation through body dissociation was not significant (*b* = .219, 95% CI [− 0.020, 0.672]; see Fig. 3A), there was a significant indirect effect of emotional abuse on emotion dysregulation through body dissociation (*b* = .386, 95% CI [0.092, 0.915]; see Fig. 3B). Results of additional models with the subscales physical neglect, physical abuse, and sexual abuse are provided in the Supplemental Material (see also Fig. S7).

## Discussion

In the present study, we investigated the mediating role of body connection as indexed by body awareness and body dissociation in the association between traumatic childhood experiences and emotion dysregulation in BPD. The current results confirmed reduced body awareness and increased body dissociation in individuals with BPD compared to HC. Importantly, we provided first evidence for a mediational role of body dissociation in the association between between traumatic childhood experiences and impaired emotion regulation.

Our findings of reduced body awareness and enhanced body dissociation in women with BPD are in line with earlier studies reporting disturbances in higher-order body representations in BPD [32, 33, 55]. The present study adds body connection to these BPD-related impairments and emphasizes the disturbed integration of bottom-up bodily signals and top-down-driven cognitive processes [26, 56].

Most importantly, our findings contribute to the understanding of the relationship between a history of traumatic childhood experiences and current deficits in emotion regulation by emphasizing the mediating role of proper body connection. Women with BPD particularly reported significantly lower body awareness and higher body dissociation compared to HC, and body dissociation was significantly related to traumatic childhood experiences, confirming previous findings in non-clinical samples [25]. A possible explanation for the non-significant association between body awareness and traumatic childhood experiences might be a more pronounced impact of traumatic childhood experiences on body dissociation. Body dissociation, as a non-pathological detachment from one’s body in an attempt to avoid adverse body experiences [25], might be regarded as a potential strategy to regulate negative emotions. Our mediation analyses provide support for this conclusion: only body dissociation was found to fully mediate the association between traumatic childhood experiences and emotion dysregulation. Importantly, this mediation effect was found even after controlling for trait dissociation as a rather general dysfunctional response to traumatic stress [29]. This is of particular importance, since dissociative experiences are common in BPD [28, 29], and more than 60% of the BPD participants in our study showed FDS scores above the suggested cutoff-score of 13 [49], indicative of pathological dissociative experiences. Furthermore, the results emphasize the differential role body dissociation might play for the development or maintenance of BPD psychopathology as compared to trait dissociation in general. In our BPD sample, the observed mediation pathway between traumatic childhood experiences and deficits in emotion regulation through body dissociation extends previous models proposing that an intact body connection plays a crucial role for emotion recognition and regulation [57, 58]. Since patients with BPD also show experiential avoidance [59], being prepared to accept bodily signals seems to be of particular relevance for the treatment of BPD. Interestingly, our additional analyses revealed a significant mediation model only for emotional abuse, but not for emotional neglect [60]. Emotional abuse, compared to emotional neglect, might more strongly affect emotion regulation capacities and learning of the regulation of one’s own emotions, which has been stated for invalidation that represents one of the most important etiological factors for impaired emotion regulation capabilities in BPD [9]. However, more studies are needed to investigate and confirm the differential role of body dissociation in associations between certain forms of early trauma and emotion dysregulation. It also needs to be noted that the supplemental results on other forms of traumatic childhood experiences need to be interpreted with care due to limited variance (e.g., 51% of the current clinical sample reported no history of sexual abuse), which may obscure a potential link between these forms of traumatic childhood experiences and emotion dysregulation.

Assessing body connection via self-report of the awareness and attention to bodily signals incorporates attentional and appraisal processes over different body modalities. Therefore, self-report assessments differ in regard to the involved mental processes, motivations, and accessible bodily processes [61]. Thus, while we found significant differences in body awareness levels using the SBC, no differences between female patients with BPD and HC on the Body Awareness Questionnaire (BAQ) [62] have been reported [63]. A possible explanation could be that the BAQ measures the attentiveness to normal non-emotional bodily processes [64], while body awareness in the SBC also incoperates the identification of links between physiological states and emotion as well as the willingness to attend to bodily signals for self-care [26]. In contrast, body dissociation describes insufficient integration of aversive bodily responses due to emotional states. Compared to general body awareness as assessed with the BAQ, body awareness and dissociation in the SBC might thus be of higher clinical importance for BPD symptomatology, which is characterized by deficits in emotion recognition and regulation. This is supported by significant and meaningful associations between body awareness and body dissociation and central BPD symptoms, such as depressiveness and anxiety, as reported in the supplement. Body connection as measured by the SBC could therefore better cover symptomatic and disorder-specific problems in women with BPD than purely perceptual ratings. It has to be noted that dissociation is a heterogeneous construct which incorporates psychoform and somatoform subtypes [35]. The supplemental results confirm a mediating role of body dissociation when general psychoform dissociation and conversion, as an estimate for somatoform dissociation, were statistically controlled for.

Body awareness as measured by the SBC can be used as a proxy for interoceptive awareness [26, 56], i.e., the processing and perception of signals from the inner body. Interoception is a multifaceted process, ranging from the preconscious cortical representation of afferent signals to the conscious awareness of bodily signals [65, 66]. According to this perspective, our findings of lower body awareness and higher body dissociation are in line with reduced heartbeat-evoked potentials (HEPs) as a cortical interoceptive marker for cardiac signals and a corresponding association with deficient emotion regulation capabilities in patients with BPD [19, 20]. However, in the domain of heartbeat perception, normal interoceptive accuracy has been reported for BPD [67]. This apparent contradiction between the cortical representation, the self-evaluation of one’s own body connection, and objective performance has not been experimentally clarified yet. A possible explanation could be that patients with BPD might be able to compensate for a reduced cortical representation of afferent cardiac signals by an enhanced attention level, while still having reduced trust in their own perception abilities due to heightened random noise in the cortical representation of interoceptive signals [16]. Similarly, patients with BPD show reduced confidence in emotion perception [68, 69]. An emotion regulation task [70, 71] could be used to experimentally investigate emotion regulation deficits in patients with BPD and its association to body connection. The convergence between objective performance in interoceptive tasks and higher-order representations of one’s own interoceptive abilities [66] could be used as more objective indicators of body connection and help to shed light on the inconsistent effects of previous studies [19, 67].

Interoception has been suggested as a transdiagnostic process for the perception and regulation of emotions [72–74]. As a basic psychobiological process, it overlaps with the cognitive systems constructs delineated in the Research Domain Criteria (RDoC) matrix. The RDoC matrix is a theoretical framework of the U.S. National Institute of Mental Health, in which varying degrees of dysfunction in general psychological/biological systems are dimensionally conceptualized. Body connection and interoception overlap with somatosensory perception within the cognitive systems construct. Investigating body connection and interoceptive processes and their relationship to other systems might further corroborate the importance body connection might play in linking a history of traumatic childhood experiences to current deficits in emotion regulation.

Although our cross-sectional mediation analyses do not allow for causal interpretations, there is evidence that strengthening body connection has positive effects on BPD symptomatology. Mindfulness is an important aspect of psychotherapies such as Dialectical Behavioral Therapy [9], an effective treatment for BPD [75]. Furthermore, recent results for a training intervention specifically targeting interoceptive skills, that is, the Mindful Awareness in Body-oriented Therapy [76], show beneficial effects on emotion regulation capabilities in traumatized women with substance use disorder [77, 78] and could therefore also be of interest for the treatment of BPD.

Several limitations of our study should be taken into account. First, we only investigated women and results may not directly be transferred to men, since sex differences have been reported for body awareness, but not for body dissociation [26]. Another major limitation is that we cannot draw any conclusions about healthy women as the HC group was excluded from the mediation analysis due to insufficient variance in core variables. Similarly, we cannot draw any conclusions on clinical groups other than BPD. Future studies examining individuals with traumatic childhood experiences without or with other mental disorders are therefore an important next step. The association between an altered body connection and the risk of BPD diagnosis was not the scope of the current mediation analysis and needs to be adressed in prospective studies. Furthermore, body connection was solely measured by self-report and studies including experimental or physiological data on body connection as well as longitudinal data are needed. Studies across the lifespan (including those accompanying patients with BPD from the current to the remitted stage) as well as interventional studies targeting body connection could help to evaluate the predictive value of our results.

## Conclusion

Traumatic childhood experiences represent an important risk factor for the development of emotion dysregulation, a core symptom of BPD. The present findings suggest elevated body dissociation as an important mediator in the association between traumatic childhood experiences and emotion dysregulation, thus confirming the importance of interventions targeting the improvement of the body connection in BPD.

## Supplementary Information

**Additional file 1.**

## Data Availability

The dataset supporting the conclusion of this article are held by the authors and will be made available upon reasonable request.

## Abbreviations

BAQ: Body Awareness Questionnaire
BDI: Beck Depression Inventory
BPD: Borderline personality disorder
BSL-23: Short version of the Borderline Symptom List
CTQ: Childhood Trauma Questionnaire
DERS: Difficulties in Emotion Regulation Scale
FDS: German adaptation of the Dissociative Experience Scale (DES)
HC: Healthy controls
IPDE: International Personality Disorder Examination
RDoC: Research Domain Criteria
SBC: Scale of Body Connection
SSRI: Selective serotonin reuptake inhibitor
STAI: State-Trait-Anxiety Inventory
