# Supplementary material for: Body connection mediates the relationship between traumatic childhood experiences and impaired emotion regulation in borderline personality disorder
Source: Borderline Personal Disord Emot Dysregul. 2021 May 17;8:17. doi: 10.1186/s40479-021-00157-7 (PMC8127297; doi:10.1186/s40479-021-00157-7)
Supplement: Supplementary file 1 — Additional file 1. [file 40479_2021_157_MOESM1_ESM.docx]

**Supplementary Information**

**Supplementary results**

**Scale of Body Connection group differences**

A Kruskal-Wallis test was used to compare the groups (current BPD, remitted BPD, HC) regarding body awareness and body dissociation. Both body awareness (*H*(2) = 28.28, *p* < .001, Fig. S1) and body dissociation (*H*(2) = 132.60, *p* < .001, Fig. S2) became significant. Post hoc pairwise comparisons (Bonferroni-corrected) revealed that patients with current BPD showed significantly lower levels of body awareness (*M* = 2.13; *SD* = 0.60; *Mdn* = 2.17; *IQR* = 0.85) than healthy controls (*M* = 2.59; *SD* = 0.58; *Mdn* = 2.63; *IQR* = 0.75; *p_Bonf_* < .001, *r* = 0.36) and remitted patients (*M* = 2.59; *SD* = 0.56; *Mdn* = 2.67; *IQR* = 0.53; *p_Bonf_* = .006, *r* = 0.29), whereas healthy controls did not differ from remitted patients (*p_Bonf_* = 1.00, *r* = 0.02). Patients with current BPD showed significantly higher levels of body dissociation (*M* = 1.90; *SD* = 0.66; *Mdn* = 1.88; *IQR* = 1.00) than healthy controls (*M* = 0.53; *SD* = 0.37; *Mdn* = 0.50; *IQR* = 0.44; *p_Bonf_* < .001, *r* = 0.84) and remitted patients (*M* = 1.06; *SD* = 0.47; *Mdn* = 1.00; *IQR* = 0.88; *p_Bonf_* = .002, *r* = 0.32), and HC also differed from remitted patients (*p_Bonf_* = .006, *r* = 0.29).


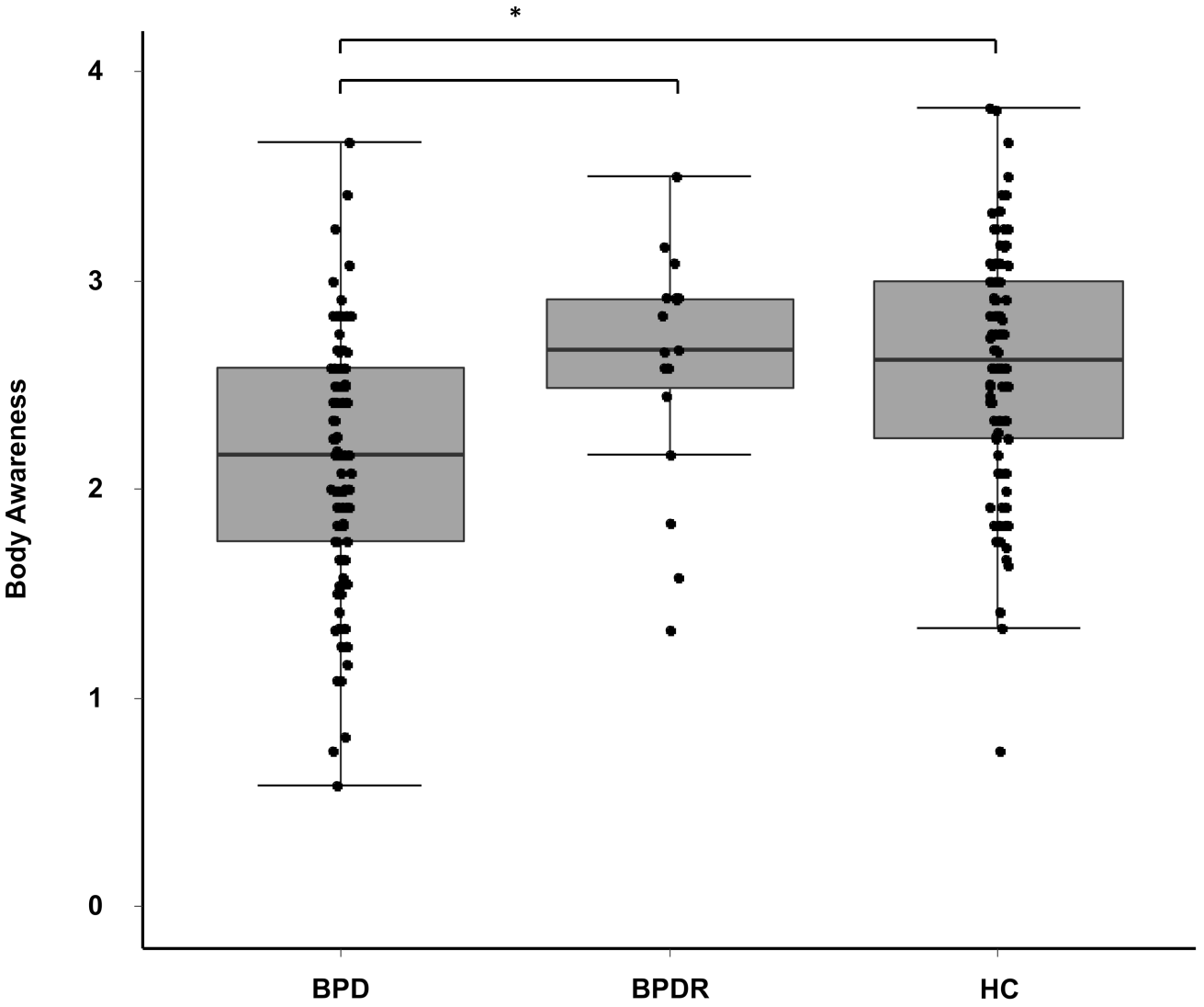


**Supplementary Fig. S1.** Body awareness for patients with current borderline personality disorder (BPD), patients with BPD in remission (BPDR) and healthy controls (HC). Indicated is the median and the interquartile range. Individual values above or below than 1.5 x interquartile range (i.e., the extent of whiskers) are considered outliers. * *p* < .01

**
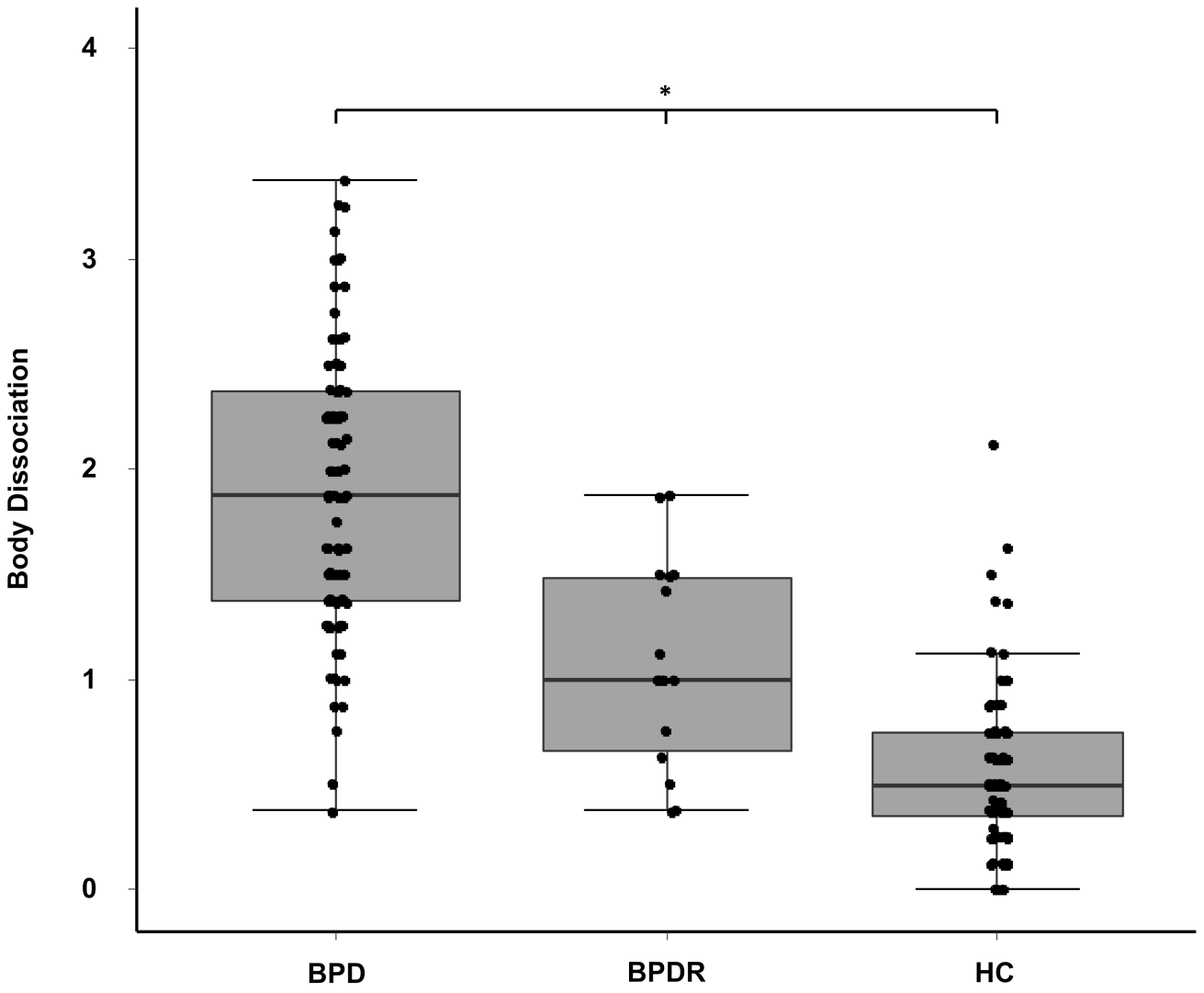
**

**Supplementary Fig. S2.** Body dissociation for patients with current borderline personality disorder (BPD), patients with BPD in remission (BPDR) and healthy controls (HC). Indicated is the median and the interquartile range. Individual values above or below than 1.5 x interquartile range (i.e., the extent of whiskers) are considered outliers. * *p* < .01

**Correlation analyses**

Further correlation analyses between body awareness and body dissociation as well as common symptoms of patients with BPD (corrected for multiple testing) were carried out which revealed that body awareness was negatively and body dissociation was positively related to borderline symptom severity, depressiveness, and trait anxiety (for statistical values see Table S1). After controlling for trait dissociation, only the associations between body awareness and depressiveness as well as body dissociation and borderline symptom severity and depressiveness remained statistically significant (see Table S1).

**Supplementary Table S1.** Correlations between body awareness, body dissociation, and common clinical symptoms of patients with BPD.

|  | Body awareness | Body dissociation | Difference (z) |
| --- | --- | --- | --- |
| Borderline symptom severity (BSL) | -.255*  [-.191] | .476***  [.293**] | -4.98*** |
| Depressiveness (BDI) | -.289**  [-.242*] | .391***  [.251*] | -4.55*** |
| Trait anxiety (STAI) | -.240*  [-.177] | .333***  [.114] | -3.79*** |

Based on Spearman’s correlations, corrected for multiple testing. The z values denote significant differences between correlation co-efficients for body awareness and body dissociation. Partial correlations controlling for trait dissociation (FDS) are denoted by square brackets. Abbreviations: BDI, Beck Depression Inventory; BSL, short version of the Borderline Symptom List; FDS, German adaptation of the Dissociative Experience Scale (DES); State-Trait-Anxiety Inventory. * *p* < .05. ** *p* < .01. *** *p* < .001

**Mediation analysis**

In addition to the reported mediation analysis with body awareness, body dissociation and trait dissociation as parallel mediators (cf. Model 2 in the manuscript) within the BPD group, we computed the analysis in the whole sample including the healthy controls (*N* = 208; see Fig. S3A) and in a combined BPD-HC sample including only participants who reported traumatic childhood experiences (i.e., a CTQ total score > 25; *N* = 188; BPD: *n* = 111, HC: *n* = 77; see Fig. S3B). In both samples, significant indirect effects emerged for body dissociation (Fig. S3A: *b* = .479, 95% CI [0.304, 0.691], Fig. S3B: *b* = .496, 95% CI [0.310, 0.722]) and trait dissociation (Fig. S3A: *b* = .154, 95% CI [0.030, 0.328], Fig. S3B: *b* = .147, 95% CI [0.024, 0.316]). The total effects of early traumatization on deficits in emotion regulation were significant (Fig. S3A: *b* = 1.32, *p* < .001, Fig. S3B: *b* = 1.24, *p* < .001) and also the direct effects of early traumatization on deficits in emotion regulation after the inclusion of the mediators were significant (Fig. S3A: *b* = .673, *p* < .001, adjusted robust *R^2^* = .737; Fig. S3B: *b* = .579, *p* = .003, adjusted robust *R^2^* = .488), suggesting that body dissociation and trait dissociation partly mediated the relationship between early traumatization and deficits in emotion regulation in the whole sample and in the subsample with traumatic childhood experiences. Therefore, the obtained result of a mediation effect via body dissociation in the BPD group was partly confirmed. It must be noted however, that the BPD group and the healthy controls differed in all included variables and form an inhomogeneous global sample which is why these results have to interpreted with caution. According to the procedure for the main manuscript, the following results base on the BPD-only group.

We additionally computed Model 1 as desribed in the main text separately for body awareness (see Fig. S4A) and body dissociation (see Fig. S5A) as mediators. Other dissociative experiences (as measured with the FDS) (cf. Model 2 in the manuscript) were added as a parallel mediator (see Fig. S4B and Fig. S5B). There was no significant indirect effect of early traumatization (as measured with the CTQ total score) on emotion dysregulation (as measured with the DERS total score) through body awareness (Fig. S4A: *b* = .043, 95% CI [-0.012, 0.194]; Fig.S4B: *b* = .036, 95% CI [-0.013, 0.171] and *b* = .072, 95% CI [-0.001, 0.209] for trait dissociation) in the patient group. However, the indirect effects of early traumatization on deficits in emotion regulation through body dissociation were significant (Fig. S5A: *b* = .163, 95% CI [0.045, 0.351]; Fig. S5B: *b* = .131, 95% CI [0.031, 0.320] and *b* = .043, 95% CI [-0.004, 0.155] for trait dissociation). In both models with body dissociation as mediator, the total effect of early traumatization on deficits in emotion regulation was significant (Fig. S5A: *b* = .461, *p* = .005, Fig. S5B: *b* = .441, *p* = .009) and the direct effect of early traumatization on deficits in emotion regulation after the inclusion of body dissociation was not significant (Fig. S5A: *b* = .298, *p* = .058, adjusted robust *R^2^* = .191; Fig. S5B: *b* = .267, *p* = .113, adjusted robust *R^2^* = .199), suggesting that body dissociation fully mediated the relationship between early traumatization and deficits in emotion regulation also in absence of body awareness as a parallel mediator. The separate mediation analyses therefore confirmed the results reported in the manuscript.

In addition, we computed separate mediation analyses with the conversion scale of the FDS and the DES as parallel mediators as estimates of somatoform and psychoform dissociation (see Fig. S6A and Fig. S6B), respectively. In both models, significant indirect effects emerged only for body dissociation (Fig. S6A: *b* = .149, 95% CI [0.040, 0.328], Fig. S6B: *b* = .118, 95% CI [0.024, 0.304]). The significant direct effect in the model including the DES (Fig. S6B: *b* = .253, *p* = .014) suggests a partial mediation when trait dissociation without inclusion of conversion symptomes was entered as a parallel mediator. Taken together, the additional mediation analyses further confirmed the results reported in the manuscript.

We additionally report the statistics for the mediations models with the three CTQ subscales *physical neglect*, *physical abuse* and *sexual abuse*. There was a significant indirect effect through body dissociation in case of the scale physical neglect (*b* = .471, 95% CI [0.035, 1.315]) but not in the case of physical abuse (*b* = -.103, 95% CI [-0.672, 0.402]). Figure S7 shows the explorative mediation analysis with the CTQ subscale *sexual abuse*. There was a significant indirect effect of sexual abuse on emotion regulation through both body dissociation (*b* = .678, 95% CI [0.208, 1.449]) and trait dissociation (*b* = .335, 95% CI [0.026, 0.930]). Neither the total effect of early sexual abuse on deficits in emotion regulation (*b* = .707, *p* = .156), nor the direct effect was significant (*b* = -.447, p = .363). The obtained pattern suggests that no direct relationship between early sexual abuse (as measured by the CTQ) and emotion regulation deficits (as measured by the DERS) existed in the current sample (*b* =.087, *p* = .180), whereas early sexual abuse was associated with both mediators which in turn were associated with emotion regulation, thereby representing an indirect mediation.


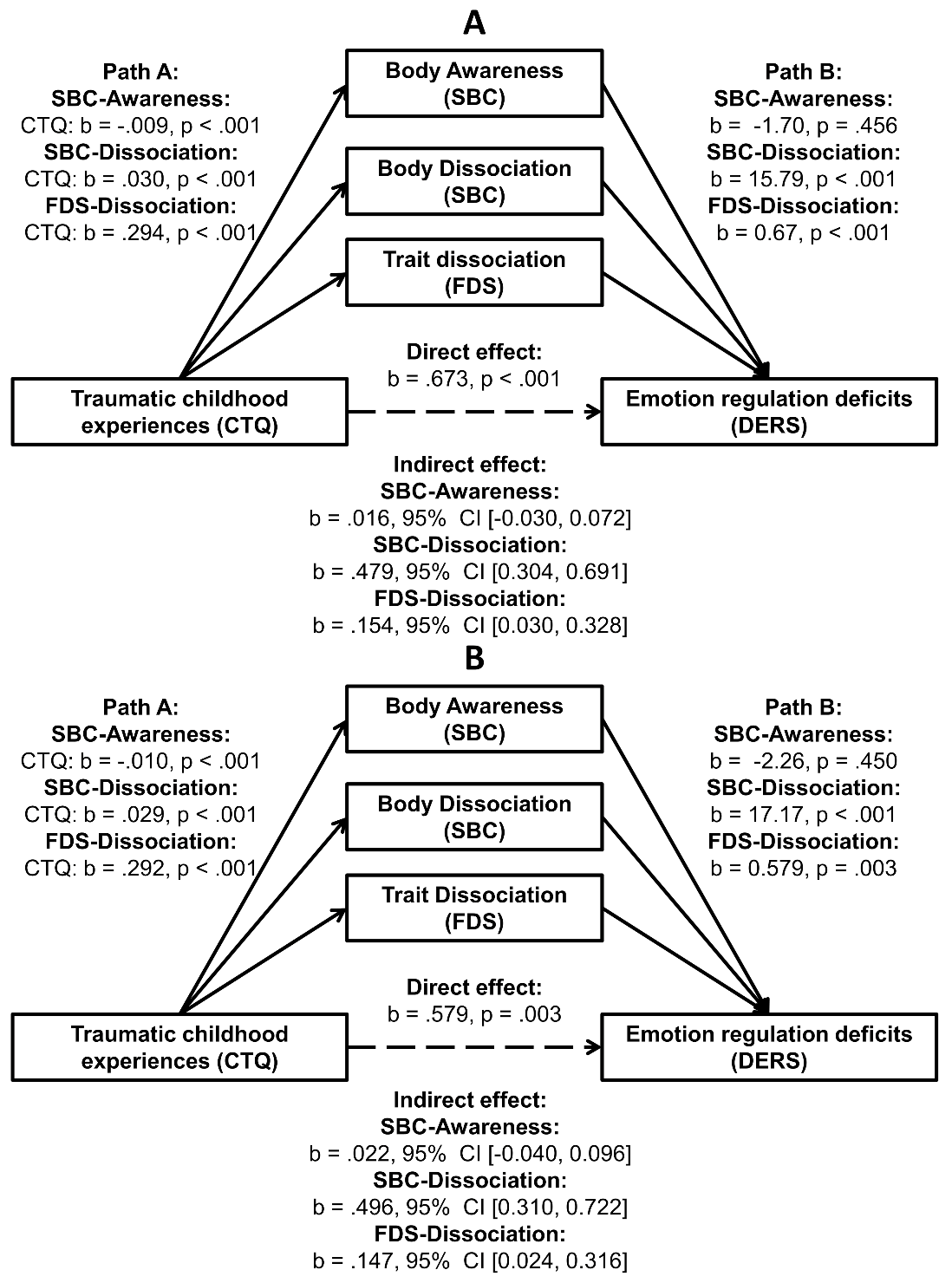


**Supplementary Fig. S3.** Parallel mediation of early traumatization and emotion regulation deficits by *body awareness* and *body dissociation*. Model A: Whole sample (*N* = 208). Model B: Subsample who reported traumatic childhood experiences (*N* = 188; *BPD: n = 111, HC: n = 77*; CTQ-score > 25). *Path A* represents the effect of early traumatization on each mediator; *Path B* represents the combined effects of each mediator on emotion regulation deficits; the *direct effect* represents the effect of early traumatization on emotion regulation deficits, while keeping levels of the mediators constant; the *indirect effect* represents the combined effect of path A and path B and therefore the mediation. The *total effect* (not shown here) represents the combined indirect and direct effects. Significance inferences at the 0.05 α level for *indirect effects* are based upon the notion whether confidence intervals include zero. *Note:* Trait dissociation included as parallel mediator. *Abbreviations:* CTQ, Childhood Trauma Questionnaire; DERS, Difficulties in Emotion Regulation Scale; FDS, German adaptation of the Dissociative Experience Scale; SBC; Scale of Body Connection


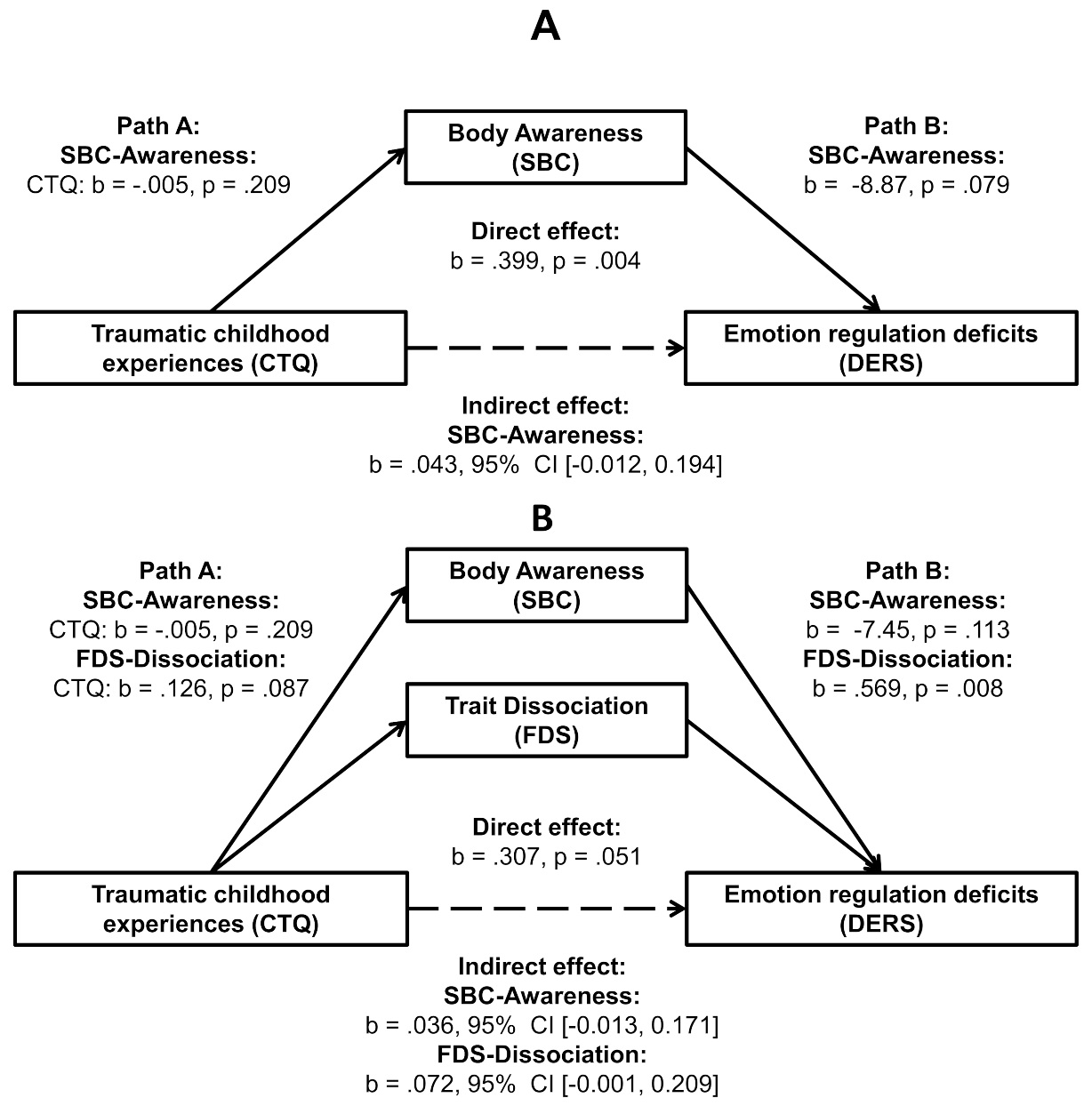


**Supplementary Fig. S4.** Mediation of early traumatization and emotion regulation deficits by body awareness in women with BPD. *Path A* represents the effect of early traumatization on each mediator; *Path B* represents the combined effects of each mediator on emotion regulation deficits; the *direct effect* represents the effect of early traumatization on emotion regulation deficits, while keeping levels of the mediators constant; the *indirect effect* represents the combined effect of path A and path B and therefore the mediation. The *total effect* (not shown here) represents the combined indirect and direct effects. Significance inferences at the 0.05 α level for *indirect effects* are based upon the notion whether confidence intervals include zero. *Note:* Trait dissociation included as parallel mediator in Model B. *Abbreviations:* CTQ, Childhood Trauma Questionnaire; DERS, Difficulties in Emotion Regulation Scale; FDS, German adaptation of the Dissociative Experience Scale; SBC; Scale of Body Connection

**
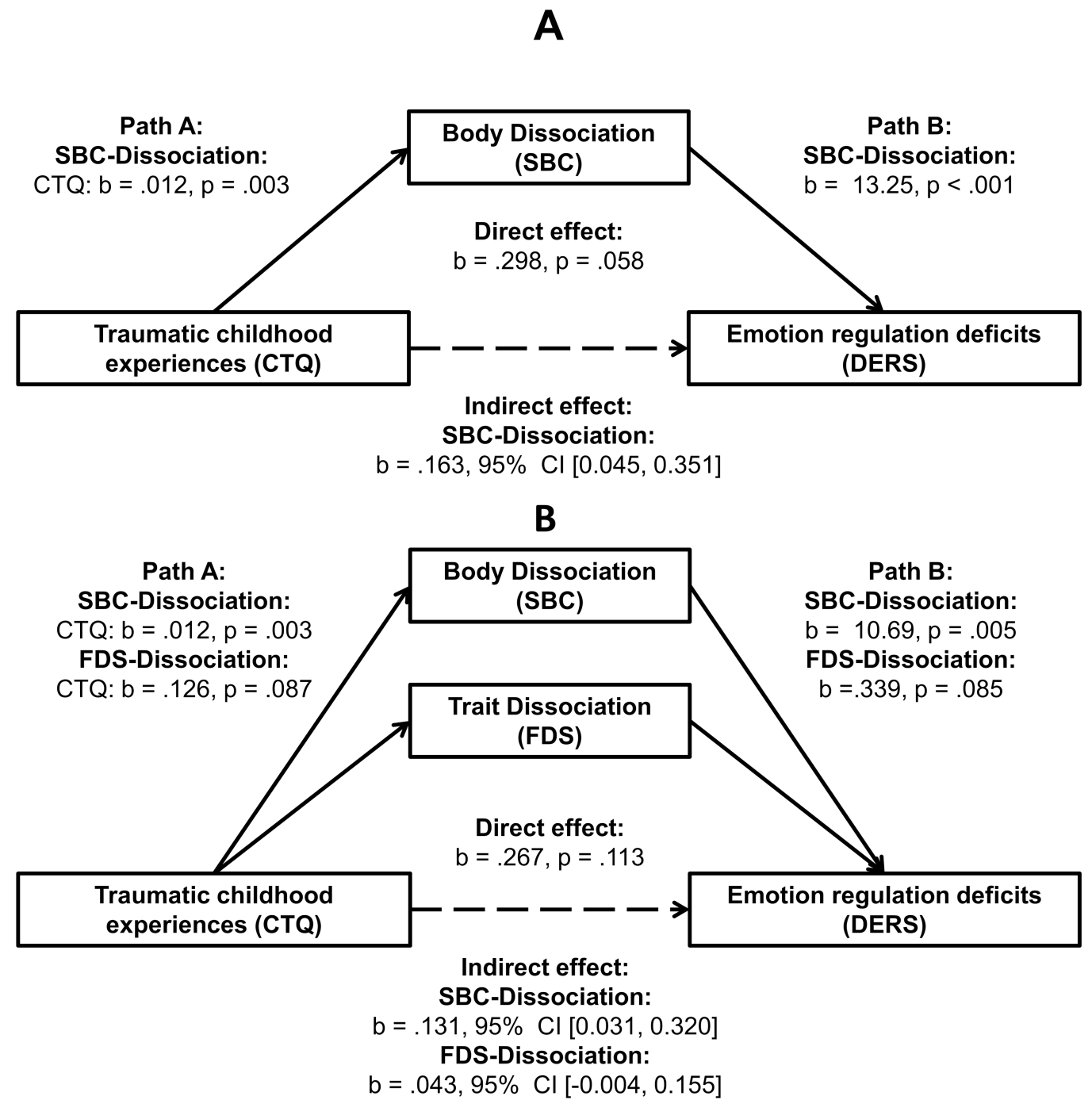
**

**Supplementary Fig. S5.** Mediation of early traumatization and emotion regulation deficits by body dissociation in women with BPD. *Path A* represents the effect of early traumatization on each mediator; *Path B* represents the combined effects of each mediator on emotion regulation deficits; the *direct effect* represents the effect of early traumatization on emotion regulation deficits, while keeping levels of the mediators constant; the *indirect effect* represents the combined effect of path A and path B and therefore the mediation. The *total effect* (not shown here) represents the combined indirect and direct effects. Significance inferences at the 0.05 α level for *indirect effects* are based upon the notion whether confidence intervals include zero. *Note:* Trait dissociation included as parallel mediator in Model B*. Abbreviations*: CTQ, Childhood Trauma Questionnaire; DERS, Difficulties in Emotion Regulation Scale; FDS, German adaptation of the Dissociative Experience Scale; SBC; Scale of Body Connection


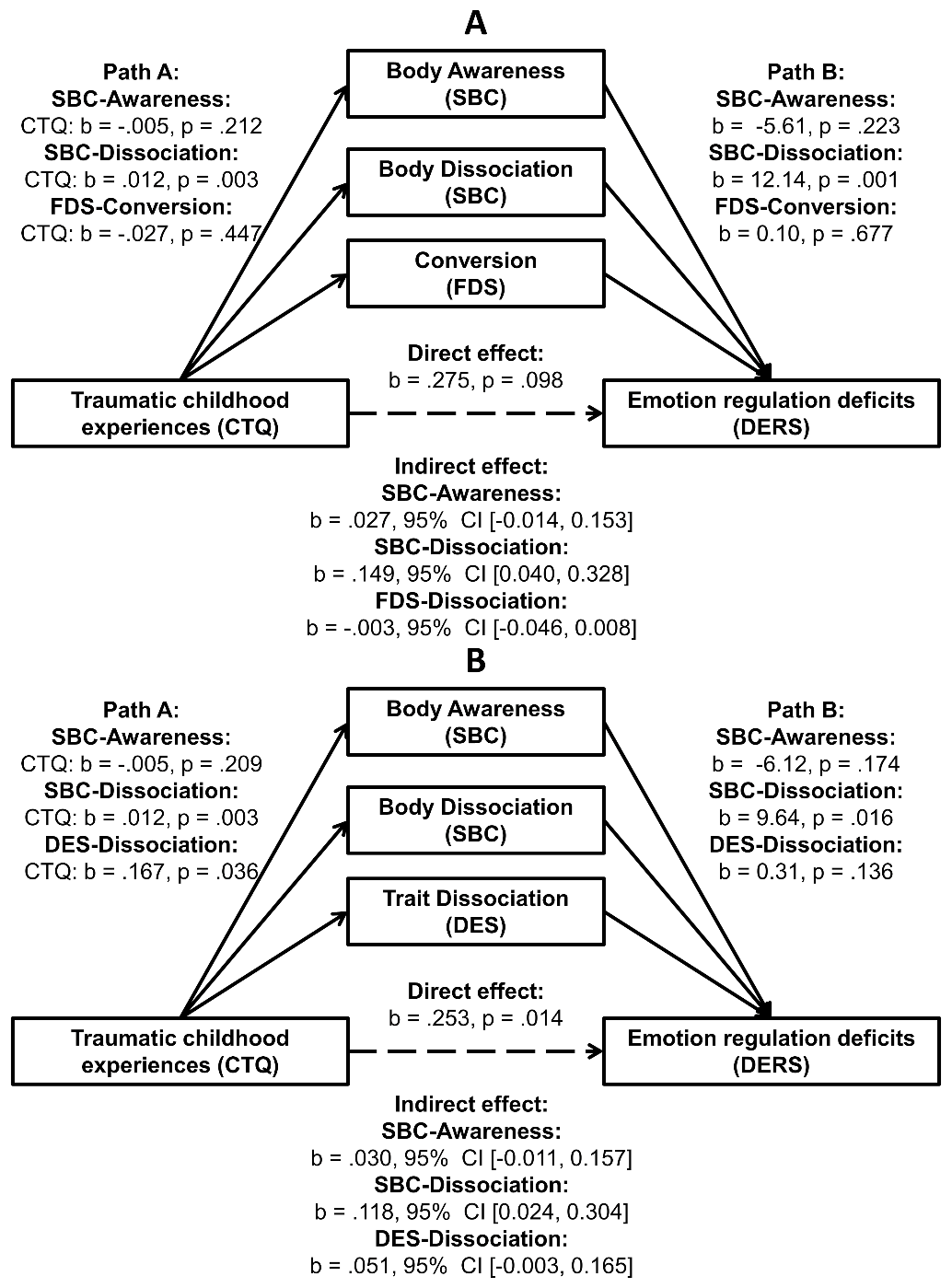


**Supplementary Fig. S6.** Parallel mediation of early traumatization and emotion regulation deficits by body awareness, body dissociation and dissociation subtypes in women with BPD. *Path A* represents the effect of early traumatization on each mediator; *Path B* represents the combined effects of each mediator on emotion regulation deficits; the *direct effect* represents the effect of early traumatization on emotion regulation deficits, while keeping levels of the mediators constant; the *indirect effect* represents the combined effect of path A and path B and therefore the mediation. The *total effect* (not shown here) represents the combined indirect and direct effects. Significance inferences at the 0.05 α level for *indirect effects* are based upon the notion whether confidence intervals include zero. *Abbreviations:* CTQ, Childhood Trauma Questionnaire; DERS, Difficulties in Emotion Regulation Scale; DES, Dissociative Experience Scale, FDS, German adaptation of the Dissociative Experience Scale; SBC; Scale of Body Connection


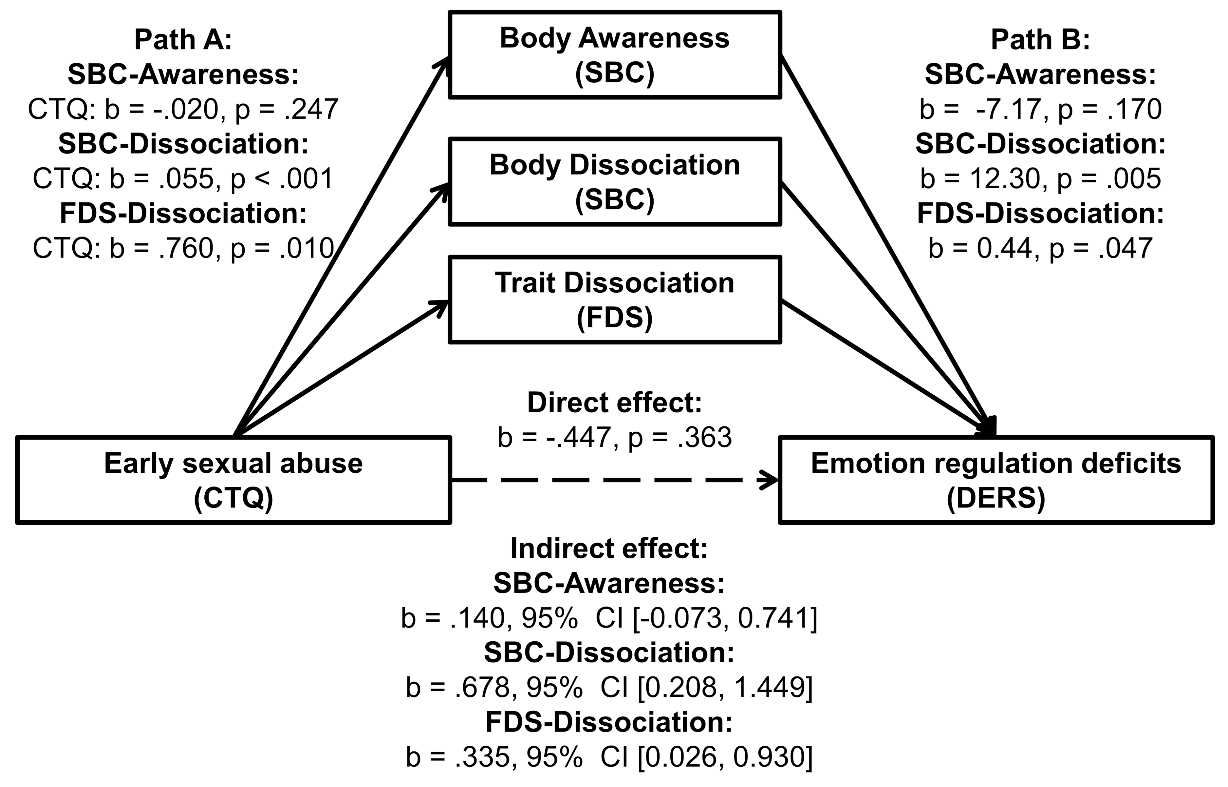


**Supplementary Fig. S7.** Mediation of early sexual abuse and emotion regulation deficits by body awareness, body dissociation and trait dissociation in women with BPD. *Path A* represents the effect of early traumatization on each mediator; *Path B* represents the combined effects of each mediator on emotion regulation deficits; the *direct effect* represents the effect of early traumatization on emotion regulation deficits, while keeping levels of the mediators constant; the *indirect effect* represents the combined effect of path A and path B and therefore the mediation. The *total effect* (not shown here) represents the combined indirect and direct effects. Significance inferences at the 0.05 α level for *indirect effects* are based upon the notion whether confidence intervals include zero. *Note:* Trait dissociation included as parallel mediator in Model B. *Abbreviations:* CTQ, Childhood Trauma Questionnaire; DERS, Difficulties in Emotion Regulation Scale; FDS, German adaptation of the Dissociative Experience Scale; SBC; Scale of Body Connection

**Demographic, clinical, diagnostic and self-reported information**

Current and lifetime comorbid mental disorders as well as regular SSRI intake are given in Table S2. Demographic, clinical, and self-reported data are depicted in Table S3. The three groups did not differ in age (BPD: *M* = 29.68, *SD* = 7.70; BPDR: *M* = 30.17, *SD* = 5.86; HC: *M* = 28.01, *SD* = 7.58; *F*_(2,205)_ = 1.43, *p* = .241).

**Supplementary Table S2.** Comparison of patients with borderline personality disorder (BPD), borderline personality disorder in remission (BPDR) and healthy controls (HC) in terms of the prevalence of comorbid psychiatric diagnoses and regular intake of SSRI.

|  | BPD  (*n* = 94) | BPDR  (*n* = 18) | HC  (*n* = 96 ) |
| --- | --- | --- | --- |
| Current comorbid Diagnoses | Number of current (lifetime) diagnoses | Number of current (lifetime) diagnoses | Number of current (lifetime) diagnoses) |
| Affective disorders | 26 (75) | 1 (15) | 0 (0) |
| Posttraumatic stress disorder | 28 (40) | 0 (7) | 0 (0) |
| Anxiety disorders | 45 (52) | 2 (10) | 0 (0) |
| Body dismorphic disorder | 1 (1) | 0 (0) | 0 (0) |
| Eating disorders | 18 (46) | 0 (9) | 0 (0) |
| Personality disorders |  |  |  |
| Antisocial | 1 (3) | 0 (1) | 0 (0) |
| Avoidant | 30 (32) | 0 (2) | 0 (0) |
| Current regular intake of SSRI (No.) | Number  12 | Number  1 | Number  0 |

*Abbreviations:* BPD, patients with borderline personality disorder; BPDR, patients with borderline personality disorder in remission; HC, healthy controls; SSRI, selective serotonin reuptake inhibitor.

**Supplementary Table S3.** Clinical and self-reported data of patients with borderline personality disorder (BPD), patients with borderline personality disorder in remission (BPDR) and healthy controls (HC).

| Construct | Mean+SD  (Mdn; IQR) | | | *H* Value | *p* Value*^a^* | *P* Value*^b^* | | |
| --- | --- | --- | --- | --- | --- | --- | --- | --- |
|  | **BPD**  **(*n* = 94)** | **BPDR**  **(*n* = 18)** | **HC**  **(*n* = 96 )** |  |  | **BPD vs BPDR** | **BPD vs**  **HC** | **BPDR vs**  **HC** |
| BPD dimensional Score (IPDE) | 14.28+1.82  (15.00; 2.00) | 4.17+2.71  (4.50; 4.00) | 0.09+0.46  (0.00; 0.00) | 180.92 | <.001 | <.001 | <.001 | .002 |
| Borderline symptoms  (BSL-23 Score) | 1.48+0.75  (1.54; 1.28) | 0.51+0.48  (0.43; 0.42) | 0.12+0.19  (0.04; 0.17) | 145.38 | <.001 | .002 | <.001 | .002 |
| Depressiveness  (BDI) | 21.05+9.55  (21.00; 14.00) | 7.67+5.28  (8.50; 9.00) | 2.10+2.93  (1.00; 3.00) | 143.91 | <.001 | .001 | <.001 | .009 |
| Trait-Anxiety (STAI) | 62.51+7.44  (63.00; 10.25) | 41.78+9.23  (42.50; 11.50) | 31.85+7.21  (32.00; 9.00) | 154.96 | <.001 | <.001 | <.001 | .039 |
| Dissociation (FDS) | 19.70+11.84  (18.52; 15.34) | 8.48+5.75  (7.95; 8.98) | 3.42+3.65  (2.27; 3.64) | 112.15 | <.001 | .009 | <.001 | .008 |
| Traumatic childhood experiences  (CTQ) | 62.60+18.65  (63.00; 23.25) | 53.67+15.43  (50.50; 21.20) | 30.98+7.65  (28.00; 7.50) | 125.52 | <.001 | .763 | <.001 | <.001 |
| Emotional Abuse | 17.44+5.30  (18.50; 8.00) | 14.44+5.04  (14.00; 8.00) | 6.89+2.81  (6.00; 2.75) | 128.62 | <.001 | .558 | <.001 | <.001 |
| Physical Abuse | 8.63+4.48  (7.00; 6.00) | 8.44+5.41  (7.00; 4.00) | 5.48+1.89  (5.00; 0.00) | 61.84 | <.001 | 1.00 | <.001 | <.001 |
| Sexual Abuse | 8.76+5.52  (6.00; 6.00) | 6.28+2.40  (5.00; 1.50) | 5.09+0.54  (5.00; 0.00) | 60.38 | <.001 | .132 | <.001 | .053 |
| Emotional Neglect | 17.60+5.83  (18.00; 10.00) | 15.61+5.46  (16.00; 9.00) | 7.47+3.16  (7.00; 4.00) | 110.09 | <.001 | 1.00 | <.001 | <.001 |
| Physical Neglect | 10.18+3.95  (10.00; 6.00) | 8.89+2.72  (8.50; 3.75) | 6.05+1.87  (5.00; 1.00) | 77.44 | <.001 | 1.00 | <.001 | <.001 |
| Emotional Dysregulation (DERS Score) | 130.01+19.03  (131.00; 29.25) | 83.39+19.27  (77.00; 23.75) | 65.73+14.80  (63.50; 17.00) | 150.54 | <.001 | <.001 | <.001 | .042 |

*Abbreviations:* BDI, Beck Depression Inventory; BPD, patients with borderline personality disorder; BPDR, patients with borderline personality disorder in remission; BSL-23, short version of the Borderline Symptom List; CTQ, Childhood Trauma Questionnaire; DERS, Difficulties in Emotion Regulation Scale; FDS, German adaptation of the Dissociative Experience Scale (DES); H Value, test statistic of Kruskal-Wallis test; HC, healthy controls; IPDE, International Personality Disorder Examination; IQR, interquartile range; M, mean; Mdn, median; p Value, probability value; SD, standard deviation; STAI, State-Trait-Anxiety Inventory.

*Notes: ^a^ Uncorrected for multiple testing. ^b^ Corrected for multiple testing between groups via bonferroni correction.*
